# Influenza A and B Infection in Children in Urban Slum, Bangladesh

**DOI:** 10.3201/eid1310.070368

**Published:** 2007-10

**Authors:** W. Abdullah Brooks, Pauline Terebuh, Carolyn Bridges, Alexander Klimov, Doli Goswami, Amina Tahia Sharmeen, Tasnim Azim, Dean Erdman, Henrietta Hall, Stephen Luby, Robert F. Breiman

**Affiliations:** * ICDDR,B, Dhaka, Bangladesh; † Johns Hopkins University, Baltimore, Maryland, USA; ‡ Georgia State Health Department, Atlanta, Georgia, USA; § Centers for Disease Control and Prevention, Atlanta, Georgia, USA; ¶ Centers for Disease Control and Prevention–Kenya, Nairobi, Kenya

**Keywords:** influenza, fever, pneumonia, children, urban, Bangladesh, letter

**To the Editor:** Influenza A and B viruses are associated with seasonal epidemics (*1*). Influenza is increasingly recognized as a cause of severe respiratory disease among healthy children in industrialized countries (*2*–*4*). However, little information is available from developing countries (*5*).

We assessed the contribution of influenza and other respiratory viruses to febrile respiratory illness among children enrolled at the ICDDR,B Kamalapur surveillance and intervention site (*6*) in an urban slum in Dhaka, Bangladesh. This study was reviewed and approved by the Research Review and Ethical Review Committees of ICDDR,B and the Institutional Review Board of the Centers for Disease Control and Prevention (CDC) (Atlanta, GA, USA).

Surveillance in Kamalapur has been described (*6*,*7*). Briefly, the site is divided into 7 geographic strata and subdivided into geographic clusters of 50–100 households. Eighty-six clusters were randomly selected and 5,000 households within those clusters were enrolled after written consent was obtained.

Prospective fever surveillance methodology has been described (*7*). To evaluate viral causes of febrile respiratory illness, paired (acute- and convalescent-phase) serum specimens were retrospectively selected from the surveillance period of December 6, 2000 to December 5, 2001, from patients with documented fever >38.5°C, a cough for >1 day but <4 days, and age <13 years, and who were negative for antibodies to dengue by immunoglobulin M (IgM) antibody-capture ELISA.

Sera were tested by hemagglutination inhibition (HI) for influenza A (H1N1 and H3N2) virus and B virus, and by ELISA for respiratory syncytial virus, parainfluenza virus types 1, 2, and 3, adenovirus, and human metapneumovirus at CDC by using standard methods (*8*). Acute infection was defined as IgM in serum sample or a >4-fold increase in IgG titer between acute- and convalescent-phase serum samples for noninfluenza viruses or a >4-fold increase in HI titer for influenza viruses.

Statistical analysis was performed by using Stata Statistical Software Release 8.2, 2003 (Stata Corporation, College Station, TX, USA). Continuous variables were compared by using analysis of variance. Univariate categorical analysis was conducted by using 2 × 2 tables to obtain odds ratios (ORs) and 95% confidence intervals (CIs). Multivariate analysis was conducted by using stepwise forward logistic regression and all covariates significant with a 5% precision in univariate analysis. We adjusted the model for clustering (multiple observations per patient) and tested for goodness of fit.

Of 889 patients who came to the ICDDR, B Kamalpur field clinic with fever during the surveillance period, 198 (22%) met inclusion criteria for retrospective sampling. Of these, 128 had adequate paired serum specimens for influenza testing. Only 107 (83.6%) pairs had sufficient serum remaining for testing for other viruses.

Of 128 children, 21 (16%) had acute influenza infections; 2 of these children had both influenza A and B. Overall, 10 influenza A (8 H1N1 and 2 H3N2) and 13 influenza B infections were detected (Table). Other respiratory virus infections were detected in 33 children and accounted for 35 noninfluenza virus infections (Table). One child was coinfected with both influenza A (H3N2) virus and HMPV. Seven (70%) of 10 influenza A cases occurred during April–June (pre-monsoon period), and 10 (77%) influenza B cases occurred during July–September (monsoon period).

**Table:** Virus infections detected by serologic analysis in children <13 years of age, December 2000–December 2001, Dhaka, Bangladesh*

Virus	No. infections (N = 56)
Influenza A†	8
Influenza B†	11
Influenza A and B†	2
Respiratory syncytial virus‡	2
Parainfluenza type 3‡	9
Adenovirus‡	4
Human metapneumovirus‡	20

*Virus infections were defined as a >4-fold increase in titers between acute-and convalescent-phase serum samples. †From 128 serum pairs tested ‡From 107 serum pairs tested.

Data for 107 serum pairs tested for both influenza and other viruses indicated that influenza-infected children were older than children without influenza (OR 3.1, 95% CI 1.1–9.3). Multivariate analysis indicated that only reported body pain was more common in influenza patients than in others (OR 3.3, 95% CI 1.5–7.1). Three influenza-infected children had clinical pneumonia (tachypnea defined by the World Health Organization) with crepitations.

We confirmed that influenza A and B were common causes of febrile illness among children in Dhaka. Because these infections were identified in 1 of 6 dengue-negative febrile children tested for influenza, these infections may play a substantial role in respiratory diseases in these children. Our study confirms findings of a previous hospital study (*9*) but provides additional information for nonhospitalized febrile children. Acute infections coincided with the warm pre-monsoon and monsoon periods.

Our study had several limitations. First, the surveillance system was not originally designed to identify influenza and relied on fever for specimen collection. Our retrospective selection criteria reflected the classic initial manifestations of influenza (*1*,*4*), and thus could have missed nonfebrile cases. Second, the study was not designed to reflect age distribution of children with respiratory infection, but rather those with fever and who had adequate amounts of available sera. This feature potentially biases toward older children. Third, data describe only 1 year, and patterns of illness may differ in other years. Fourth, acute infection was determined by serologic analysis. Previous studies in Bangladesh reported nutrition-related impaired immune responsiveness (*10*). Thus, some influenza-infected children who showed a nondetectable immune response may not have been included.

These findings indicate that influenza and other respiratory viruses contribute to pediatric febrile illness in urban Bangladesh. They also justify prospective surveillance to better define epidemiology and clinical findings associated with these viruses.
